# Effects of Teriparatide and Alendronate on Functional Recovery from Spinal Cord Injury and Postinjury Bone Loss

**DOI:** 10.3390/biomedicines13020342

**Published:** 2025-02-03

**Authors:** Shuai Wang, Jingliang Zhu, Yuping Feng, Yuchen Hua, Gangjun You, Jahui Su, Benchao Shi

**Affiliations:** 1 Department of Spinal Surgery, Orthopedic Medical Center, Zhujiang Hospital, Southern Medical University, Guangzhou 510280, China; ws13581270434@smu.edu.cn (S.W.); zhujingliang1997@163.com (J.Z.); hyc1434023686@163.com (Y.H.); ygj314@126.com (G.Y.); s737103437@163.com (J.S.); 2 Department of Clinical Laboratory, The Third Affiliated Hospital of Soochow University, Changzhou 213003, China; 15335726695@163.com

**Keywords:** spinal cord injury, osteoporosis, teriparatide, alendronate, bone mineral density

## Abstract

Objectives: This study evaluated the efficacy of teriparatide (TPTD) and alendronate (ALN) in mitigating bone loss, enhancing bone structure, and facilitating motor function recovery following spinal cord injury (SCI). Methods: All the rats were allocated into four groups: a sham surgery group (SHAM group), a normal saline group (SCI + NS group), a TPTD treatment group after SCI (SCI + TPTD group), and an ALN treatment group after SCI (SCI + ALN group). The Basso, Beattie, and Bresnahan (BBB) scores and gait analyses were used to assess the motor abilities of rats following SCI and the effects of treatment. HE staining, Masson’s trichrome staining, and LFB staining were performed to evaluate the extent of spinal cord tissue damage. Micro-CT was used to measure 12 bone-related parameters of the proximal tibia and create 3D images, and structural changes in the proximal tibial bone tissue were observed under a light microscope after HE staining. Results: After 12 weeks of treatment, the micro-CT data indicated that TPTD significantly increased key bone indicators, such as bone mineral density, after SCI (*p* < 0.01), whereas ALN did not significantly improve these indicators (*p* > 0.05). Compared with the SCI + NS group, the SCI + TPTD group presented significantly greater BBB scores and near-normal gait parameters (*p* < 0.05). Analyses of pathological sections revealed that TPTD significantly reduced the cavity area in the spinal cord after SCI, decreased the proportion of scar tissue, and increased the retention of neural myelin (*p* < 0.05). However, ALN had no significant effect on these indicators (*p* > 0.05). Conclusions: TPTD was more effective than ALN at mitigating bone loss and promoting motor function recovery after SCI, and it demonstrated significant advantages in reducing spinal cord damage and improving tissue structure.

## 1. Introduction

Spinal cord injury (SCI) is a serious disorder of the central nervous system that arises from trauma, tumors, degenerative spinal diseases, and other etiologies and is characterized by variable levels of sensory, motor, and autonomic dysfunction [1,2]. Globally, an estimated 2–3 million individuals live with disabilities resulting from SCI. In China, the prevalence of SCI has reached 65.15 cases per million and continues to rise, placing a significant burden on patients and healthcare systems alike [3,4].

Studies have shown that bone loss can occur as early as 4 weeks post-SCI [5,6,7]. Chronic bone loss often progresses to osteoporosis, a systemic condition characterized by reduced bone mass, deterioration of the bone microarchitecture, and increased fragility, resulting in a greater risk of fractures [8]. Approximately 5% to 34% of SCI patients experience a fracture within one year of injury, and 70% of SCI patients experience low-impact fractures during their lifetime [9]. For SCI patients with fractures caused by reduced bone mass, nonsurgical treatment is most commonly used, but it can lead to a substantial decrease in quality of life and adverse outcomes. Therefore, preventing bone mass loss after SCI is crucial.

Currently, numerous drugs are available to treat osteoporosis, including agents that promote bone formation, inhibit bone resorption, have dual actions, and those with other mechanisms, even including traditional Chinese medicine [10,11]. However, the underlying mechanisms of SCI-associated bone loss are complex and distinct from those of postmenopausal or idiopathic osteoporosis [10,12]. Therefore, whether the medications used to treat osteoporosis in postmenopausal and elderly individuals are also efficacious in treating bone mass loss after SCI in the general population deserves further study. Some studies suggest that the effectiveness of currently available anti-osteoporosis medications for treating SCI-associated bone loss is limited and does not significantly increase bone mineral density (BMD) [13,14,15,16,17].

TPTD, a recombinant fragment of human parathyroid hormone (rhPTH (1-34)), is widely used to promote bone formation [18]. Teriparatide works in the same way as endogenous PTH does, acting on renal tubular cells and osteoblasts to stimulate osteoblast activity and new bone formation [19]. TPTD has dual effects: it can stimulate osteoblasts to increase bone formation and, at high doses, can also promote bone resorption [20,21,22]. PTH exerts its osteogenic effects directly on mesenchymal stem cells, osteoblast lineage cells, osteocytes, and T cells while indirectly influencing osteoclast precursor cells and mature osteoclasts. Through these interactions, PTH activates key signaling pathways, including the Wnt, cAMP/PKA, and RANKL/RANK/OPG pathways, among others, to regulate bone remodeling and homeostasis [23].

ALN is a bisphosphonate anti-osteoporosis drug and a widely used bone resorption inhibitor. It decreases bone resorption by adhering to bone surfaces and inhibiting the production of hydroxyapatite crystals [24,25,26]. In our study, we assessed the efficacy of these two representative anti-osteoporosis drugs with different mechanisms of action in treating bone loss after SCI to determine whether they have similar effects on this form of bone loss as on primary osteoporosis, such as postmenopausal osteoporosis, and thus provide a reference for their clinical application. Recent reviews and studies have suggested that TPTD and ALN may contribute to motor function recovery following SCI. Our research aimed to further verify this hypothesis [27,28].

## 2. Materials and Methods

### 2.1. Animals and Experimental Design

Sixty female SD rats weighing approximately 250 g were selected for this study, and all the animals were housed in an SPF-grade environment and maintained under standard conditions. The experimental animals were provided by the Animal Experimentation Center of Zhujiang Hospital of Southern Medical University and were approved by the Animal Ethics Committee (No. LAEC-2023-006). The intervention began one week after the animals had acclimated. Sixty rats were chosen for this study and categorized into four groups of 15 rats each based on body weight and in vivo tibial BMD: the sham surgery group, normal saline group (SCI + NS), TPTD treatment after SCI (SCI + TPTD), and ALN treatment after SCI (SCI + ALN). TPTD ( MCE, Monmouth Junction, NJ, USA) was administered subcutaneously at 40 µg/kg 3 times weekly. ALN (GLPBIO, Montclair, CA, USA) was administered intraperitoneally at 1 mg/kg five times weekly. Drug administration began on the first day after SCI and lasted for 12 weeks. Both in vivo and ex vivo bone histomorphometry were conducted throughout the experiment. Bone density measurements were recorded in vivo before the experiment, and the rats were grouped accordingly. Samples for ex vivo bone histomorphometry were collected. The animals were euthanized via an overdose of pentobarbital sodium.

### 2.2. SCI Modeling

The SCI model was constructed using forceps to injure T10 [29]. Specifically, anesthesia was induced with isoflurane (RWD Life Science, Shenzhen, China). After initial orientation using the anatomical features of the spine, the hair was removed from the surgical area with a shaver, and the area was disinfected with iodophor. After reorientation, the skin was cut, the skin and muscles over the spine were separated, the T9–T11 vertebrae were exposed, and the T10 vertebral plate was resected. The spinal cord was then clamped with precision forceps (FST, Heidelberg, Germany) for 15 s. The sham group underwent laminectomy without spinal cord compression. The bladder was squeezed 2–3 times a day to aid in urination until normal bladder function was restored.

### 2.3. Behavioral Tests

The behavioral tests were conducted independently by two investigators, both of whom were blinded to the group assignments. The Basso, Beattie, and Bresnahan (BBB) scores were evaluated at the same time each week at weeks 0, 1, 2, 3, and 4 and continued to 12 weeks post-SCI, resulting in a total of 13 assessments. The BBB scoring system, which ranges from 0 to 21 points, provides a structured evaluation of motor function recovery across three distinct levels in a rat model [30].

Gait analysis was conducted using the CatWalk XT^®^ automated gait analysis system (Noldus, Wageningen, the Netherlands). All the rats underwent training twice per week prior to SCI. Formal testing and data collection commenced one week before SCI and were conducted at the same time each week during weeks 4, 6, 8, 10, and 12 postinjury. For the CatWalk XT^®^ automated gait analysis, the parameters were set appropriately before the data were collected and analyzed [31].

### 2.4. Tissue Preparation and Histological Staining

After 12 weeks, cardiac perfusion was performed on all the animals, followed by the collection of lower limb long bones and spinal cord tissue. The collected spinal cord tissue samples were fixed, dehydrated, embedded, sectioned, and subjected to histological staining. For HE, Masson’s trichrome, and LFB staining, the following staining kits were used: an HE staining kit (Servicebio, Wuhan, China), a Masson’s trichrome staining kit (Servicebio, Wuhan, China), and an LFB staining kit (Servicebio, Wuhan, China). Tissue damage and nerve injury were evaluated by staining. Images of the stained slides were captured using a Pannoramic MIDI II scanner (3D-HISTECH, Budapest, Hungary) and analyzed with Fiji-ImageJ software 2.3.0 (NIH, Bethesda, MD, USA).

### 2.5. Micro-CT

Micro-CT of the proximal tibial plateaus in the rats was conducted using a Quantum GX2 Micro-CT system (Revvity, Waltham, MA, USA). Images were captured at 90 kV and 88 mA with additional 0.06 mm copper and 0.5 mm aluminum filters. In vivo micro-CT scanning was performed on the rats under isoflurane anesthesia with a 72 mm field of view for 4 min, and CT images were acquired at a radiation dose of 219 mGy. For ex vivo imaging, images were captured with a 36 mm field of view and reconstructed over a 14 min acquisition time. Smaller regions of interest (ROIs) were selected on ex vivo CT scans to enhance the voxel resolution, reducing the voxel size from 72 µm to 25 µm. The scanned images were analyzed by ANALYZE 14.0 software. The following parameters were analyzed: cortical BMD (g/cm^3^), trabecular BMD (g/cm^3^), bone mineral content (BMC, mg), tissue volume (TV, mm^3^), bone tissue volume (BV, mm^3^), trabecular bone volume fraction (BV/TV, %), the bone surface area-to-volume ratio (BS/BV, 1/mm), the bone surface area-to-tissue volume ratio (BS/TV, 1/mm), the trabecular thickness (Tb.Th, μm), the trabecular number (Tb.N, 1/mm^2^), the trabecular separation (Tb.Sp, μm), the cortical bone area (Ct.Ar, mm^2^), the cortical bone thickness (Ct.Th, μm), and the medullary cavity area (Ma.Ar, mm^2^). The nomenclature and symbols used are consistent with the guidelines provided by the Nomenclature Committee on Tissue Morphology of the American Society for Bone and Mineral Research [32].

### 2.6. Statistical Analysis

The data are presented as the mean ± standard deviation (SD). The Shapiro–Wilk test and Levene’s test were employed to assess data normality and homogeneity of variance, respectively. The data were analyzed using one-way or two-way analysis of variance, followed by Tukey’s post hoc test for intergroup comparisons. All analyses were performed using GraphPad Prism (version 10.4.1; GraphPad Software, Boston, MA, USA), with a significance level of *p* < 0.05.

## 3. Results

### 3.1. TPTD Can Significantly Alleviate Bone Loss After SCI

**Figure 1:**
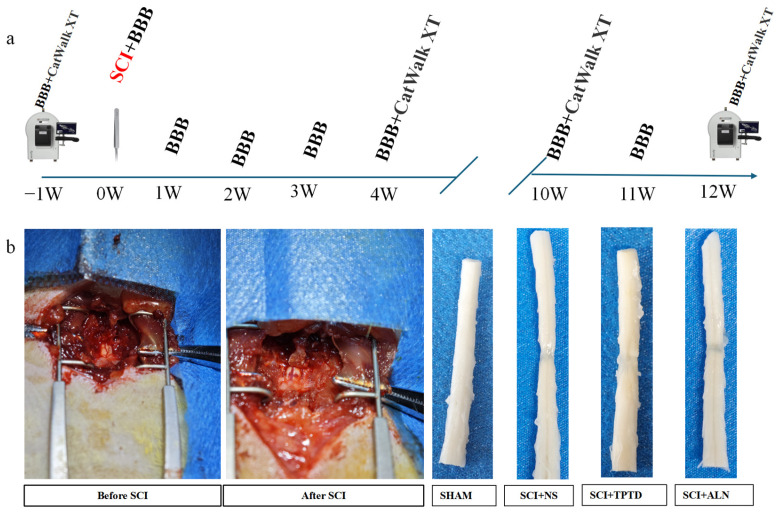
SCI model construction and evaluation. (**a**) Timeline of rat SCI model construction, behavioral assessments, and BMD measurements. (**b**) Representative images captured during model construction and gross images of the spinal cord from each group.

**Figure 2:**
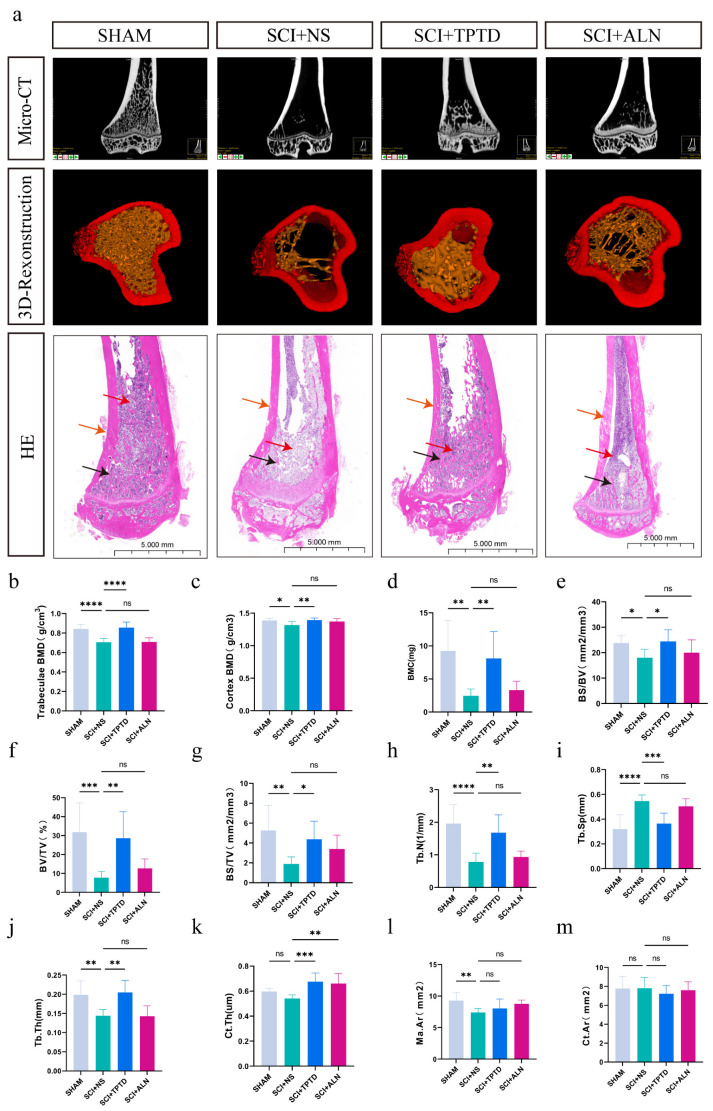
Micro-CT images of the upper part of the tibia, data analysis, and HE staining. (**a**) Representative micro-CT images of the upper part of the tibia, 3D reconstructed images, and HE-stained images. The adipose tissue in the cancellous interstitial space of the tibia is indicated by the red arrow, the cancellous bone is indicated by the black arrow, and the cortical bone is indicated by the brown arrow. (**b**–**m**) Quantitative analysis of the data obtained after 3D reconstruction and analysis of the tibia. *n* = 8/group. * *p* < 0.05, ** *p* < 0.01, *** *p* < 0.001, ****p < 0.0001, and ns, no significant difference.

An SCI model was successfully established using forceps, and the animals were treated and observed for 12 weeks postinjury (Figure 1a,b). At the end of the observation period, micro-CT and HE staining were performed on the proximal tibia. The results demonstrated a significant reduction in bone mass in the SCI + NS group. The 3D reconstructions and HE staining clearly revealed thinning of the trabeculae, which were replaced by interstitial tissues such as fat, in the SCI + NS group (planar view). Compared with saline-treated rats, TPTD- or ALN-treated rats presented significantly greater bone volume (Figure 2a). According to the micro-CT results, compared with saline-treated rats, those in the SCI + TPTD group presented notable increases in trabecular BMD, cortical BMD, BMC, BV, BV/TV%, Tb.N, Tb.Th, and Ct.Th (*p* < 0.05). Conversely, the Tb.Sp was significantly lower in the SCI + TPTD group than in the SCI + NS group (*p* < 0.05). No significant differences in Ct. Ar and Ma. Ar were compared between the TPTD and saline-treated groups (*p* > 0.05) (Figure 2b–m). In the SCI + ALN group, the Ct. Th was significantly greater than that in the SCI + NS group (*p* < 0.01), although other indicators of cortical and cancellous bone did not differ significantly, showing only a trend toward improvement (*p* > 0.05) (Figure 2b–m). Its detailed data are detailed in the Supplementary Materials (Table S1). In summary, TPTD was significantly more effective than ALN at increasing bone mass after SCI.

### 3.2. TPTD Improves the Motor Function of Rats After SCI

**Figure 3:**
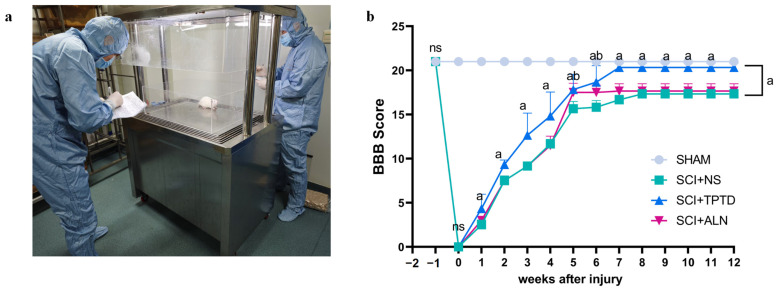
Images used for BBB scoring and data analysis. (**a**) Images of the BBB were scored by investigators in a double-blinded manner. (**b**) BBB scores. Two-way ANOVA was performed, *n* = 6/group. Subtotal a represents a significant difference between the SCI + TPTD group and the SCI + NS group (*p* < 0.05); Subtotal b represents a significant difference between the SCI + ALN group and the SCI + NS group (*p* < 0.05); ns, no significant difference.

**Figure 4:**
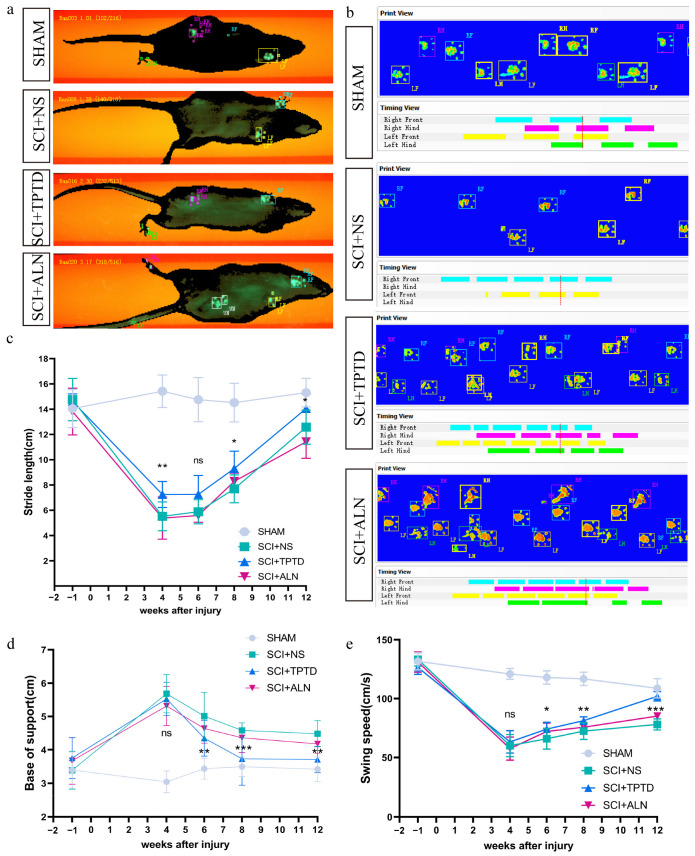
Representative images of footprints from the CatWalk gait analysis and data analysis. (**a**,**b**) Example of a CatWalk image captured from underneath an injured rat. The paws in contact with the glass catwalk are indicated by the colored frames (left front paw: yellow, left rear paw: green, right front paw: light blue, and right rear paw: magenta). (**c**–**e**) Quantitative analysis of three parameters: stride length, swing speed and base of support. *n* = 12/group. * *p* < 0.05, ** *p* < 0.01, ***p < 0.001, and ns, no significant difference.

In this study, motor function recovery in rats post-SCI was evaluated using BBB scores and gait analysis. Starting from the first week postinjury, the BBB scores of the SCI + TPTD group were significantly greater than those of the SCI + NS group, and this trend persisted until the end of the 12-week study (*p* < 0.05). In the fifth and sixth weeks after SCI, the BBB scores of the SCI + ALN group were significantly greater than those of the untreated group (*p* < 0.05) (Figure 3a,b). Gait analysis revealed that stride length and swing speed were greater in the TPTD-treated group than in the SCI + NS group (*p* < 0.05). Additionally, the distance between the two hind paws (base of support) was considerably smaller in the TPTD group than in the untreated group (*p* < 0.05), suggesting that motor function in the TPTD group improved faster and to a greater extent following SCI. However, no significant differences were found in stride length, swing speed, or base of support between the ALN and untreated groups, although a trend toward improvement was observed (*p* > 0.05) (Figure 4a–e). We speculated that TPTD may promote spinal cord repair after SCI, whereas the effects of ALN require further investigation. Our experimental findings do not support the hypothesis that ALN significantly influences spinal cord repair in rats after SCI.

### 3.3. TPTD Significantly Ameliorates Histopathological Changes After SCI

**Figure 5:**
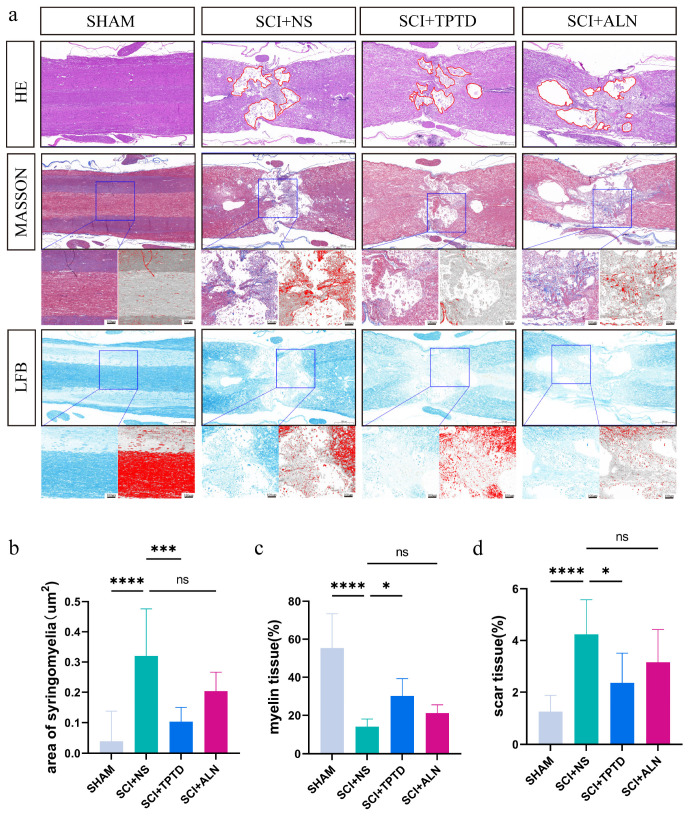
Pathological staining of spinal cord tissue sections after SCI and quantitative analysis of the relevant parameters. (**a**) From top to bottom: HE staining, Masson’s trichrome staining, and LFB staining. In HE staining, red irregular circles mark spinal cord cavities; red spots in Masson staining represent collagen fibers. Red highlights in LFB stain represent myelin tissue. (**b**) Quantitative analysis of the area of the spinal cord cavity. (**c**) Analysis of the proportion of myelin. (**d**) Analysis of the proportion of scar tissue. *n* = 8/group. * *p* < 0.05, *** *p* < 0.001, **** *p* < 0.0001, and ns, no significant difference.

In this study, spinal cord tissue recovery after SCI was assessed using three distinct staining techniques. HE staining revealed that the saline group exhibited the largest area of cavitation at the SCI site, whereas the SCI + TPTD group had significantly smaller cavitation areas than did the saline group (*p* < 0.01). A decreasing trend in cavity size was also observed in the SCI + ALN group, but the difference was not statistically significant (*p* > 0.05) (Figure 5a,b). Generally, a smaller cavity area indicates a greater degree of spinal cord repair and less severe injury. The Masson’s trichrome staining results indicated that the saline group presented severe scarring and hyperplasia, whereas the TPTD group presented significantly less scar tissue at the injury site (*p* < 0.05). The ALN group also displayed a trend toward reduced scar tissue formation, although the difference was not statistically significant (*p* > 0.05) (Figure 5d). In the subacute and chronic phases of SCI, scar tissue formation obstructs spinal cord repair; hence, minimizing scar tissue is beneficial for promoting the recovery of spinal cord function [33]. The results of the LFB staining analysis indicated that the amount of myelin was significantly lower in the saline group than in the sham group (*p* < 0.05). Conversely, the SCI + TPTD group presented a greater myelin content than the saline group did (*p* < 0.05), whereas the SCI + ALN group presented greater myelin levels, although the difference was not statistically significant (*p* > 0.05) (Figure 5c). After SCI, demyelination leads to nerve cell death. Greater myelin retention implies better nerve survival, which can reflect the extent of spinal cord tissue repair after SCI to some degree [34,35].

## 4. Discussion

The number of SCI patients worldwide is increasing annually. Although most patients survive, they suffer from varying degrees of dysfunction, especially a loss of motor function [36]. Patients with SCI who survive in the long term often experience bone loss. The mechanism of bone loss after SCI is intricate and distinct from the mechanisms underlying postmenopausal osteoporosis, senile osteoporosis, and disuse osteoporosis [37,38]. Compared with ovariectomy (OVX), SCI has a more pronounced negative impact on the geometric structure of cortical bone and the microarchitecture of trabeculae at the tibial diaphysis [37]. The precise process of bone loss after SCI remains incompletely elucidated, with alterations in bone structure and activity differing on the basis of the anatomical area and the severity of the SCI.

Currently, several mechanisms, including the RANKL and Wnt signaling pathways, reduced bone metabolic activity, and decreased weight bearing, may contribute to bone mass loss following SCI. Secondary variables associated with SCI include systemic hormonal shifts, modifications in bone innervation, and impaired bone perfusion [39,40]. Currently, interventions for addressing bone loss after SCI are limited, and more effective treatments are needed. Current interventions mainly involve anti-osteoporosis drugs, including bisphosphonates, TPTD, and denosumab.

Bisphosphonate treatment is a widely used intervention for bone loss after SCI [41]. Most research indicates that drugs can mitigate bone loss in patients after SCI, but they do not significantly increase bone density and may increase the risk of atypical femoral fractures [42]. Certain studies indicate that osteogenic drugs, such as the parathyroid hormone analog TPTD, may be utilized to address bone loss following SCI [43,44]. Research on the management of bone loss following SCI typically focuses on the impact of an individual medication and lacks comparative evaluations. Rats with SCI were treated with ALN and TPTD in this study to provide a reference for clinical intervention. Our findings indicate that TPTD is substantially more efficacious than ALN in addressing bone loss during the initial phases of SCI. Although ALN tends to improve certain bone tissue parameters, these changes are not statistically significant. Our findings align with those of prior studies that focused on the use of a single drug [45], but debate persists about the impact of bisphosphonates on bone loss following SCI. Our findings indicate that bisphosphonates exert no substantial effect on bone loss in the initial phases post-SCI. We speculate that this difference could be due to the limited impact of the drug on the substantial bone loss occurring in the initial phases after SCI and the relatively short duration of the drug treatment protocol in this study.

After SCI in rats, bone resorption increases significantly, resulting in significant bone loss. Although bone formation may initially increase, it is typically inadequate to counterbalance elevated bone resorption, resulting in a net reduction in bone mass. This imbalance persists over time, and understanding the processes of bone resorption and formation is essential for developing viable treatments to mitigate bone loss post-SCI [46,47]. Although ALN inhibits bone resorption and targets the fundamental causes of bone loss following SCI, its inhibitory effects are generally weaker than those of the bone formation-promoting drug TPTD, which leads to greater improvements in all parameters than ALN does.

There are no specific medications for the treatment of SCI, and researchers have conducted numerous studies to address this situation. In the acute phase, the standard of care is surgical decompression, which enhances local blood circulation by alleviating strain on the spinal cord, thereby diminishing the severity of secondary injury [48]. Given the limited treatment options and short window of intervention during the acute phase of SCI and the fact that secondary injuries lead to severe outcomes after SCI, neuroprotective drugs that target the secondary injury process are the focus of current research, e.g., methylprednisolone, which has been widely used but is controversial because of its possible side effects and limited efficacy [49]. Other novel agents, such as antioxidants, anti-inflammatory agents, and neurotrophic factors, have shown promising results in preclinical studies [50,51,52]. In the rehabilitation phase, physiotherapy, robotic training, and neuromodulation techniques (e.g., electrical and magnetic stimulation) have become the main tools used [53]. Emerging brain–computer interface technology also offers hope for patients to recover some motor functions [54]. In recent years, stem cell therapies derived from neural stem cells, mesenchymal stem cells induced pluripotent stem cells, and hydrogel scaffolds synthesized using various biomaterials and tissue engineering techniques have made some progress in spinal cord injury repair [55,56,57].

In this study, we observed that the TPTD-treated group exhibited faster recovery of motor function following SCI, as indicated by the BBB scores and gait analysis. In contrast, no significant difference in the speed of motor function recovery was observed between the ALN and untreated groups at most time points. However, the ALN group showed some improvements in BBB scores during weeks 5 and 6 compared with those of the untreated group. Previous studies have reported that ALN can inhibit the inflammatory response after SCI, thereby improving spinal cord function after injury [27]. However, few studies have been conducted on this topic, and additional research supporting this idea is lacking. The mechanism by which TPTD improves motor function after SCI may be related to its ability to reduce blood–spinal cord barrier permeability and inhibit oxidative stress after SCI [28,58]. Some studies have shown that TPTD can promote angiogenesis by promoting the expression of ang-1, thereby improving blood–brain barrier function [59]. TPTD may work via a similar mechanism.

The significance of TPTD, as a drug that is already heavily used in the clinic, will be important if new effects can be discovered. Next, our team will carry out more in-depth research on this topic, and we have already performed single-cell sequencing using SD rat spinal cord specimens and will further verify the mechanism through in vivo and ex vivo experiments and try to carry out clinical trials to verify it.

Some aspects of this study could be improved. The SCI model we chose is different from the percussion SCI model and the transection SCI model, and the method we used to model SCI may have had an effect on bone mass in some animals due to premature movement. Considering the duration of drug intervention and the time needed for motor recovery after SCI, we propose using the transection method to sever the spinal cord completely, extending the drug administration period, and continuously monitoring changes in bone activity during the drug administration period using an in vivo micro-CT instrument. In vivo CT imaging requires advanced equipment and a long scanning time, which poses a significant challenge for researchers. Furthermore, rat serum may be obtained at various intervals throughout the observation period to evaluate the levels of bone production and resorption indicators, as well as to detect nuanced alterations in bone metabolism.

## 5. Conclusions

This study demonstrated that TPTD can improve bone loss after SCI and that ALN has no significant effect on bone loss after SCI. Therefore, TPTD is significantly better than ALN for early intervention of bone loss in SCI. In addition, this study unexpectedly revealed that TPTD can improve the repair of SCI and facilitate the return of motor function. Conversely, ALN did not enhance the repair of SCI. Additional clinical trials employing TPTD to confirm its effectiveness in treating SCI are essential and will have considerable importance in future research.

## Data Availability

Data are contained within the article and Supplementary Materials.

## Supplementary Materials

The following supporting information can be downloaded at: https://www.mdpi.com/article/10.3390/biomedicines13020342/s1, Table S1: Indicators of bone parameters associated with the proximal tibia.
