# Supplementary material for: Effects of Teriparatide and Alendronate on Functional Recovery from Spinal Cord Injury and Postinjury Bone Loss
Source: Biomedicines. 2025 Feb 3;13(2):342. doi: 10.3390/biomedicines13020342 (PMC11852434; doi:10.3390/biomedicines13020342)
Supplement: Supplementary file 1 [file biomedicines-13-00342-s001.zip › biomedicines-3386388-supplementary.pdf]

Table S1. Indicators of bone parameters associated with the proximal tibia（**n** =8，$\bar{x} \pm s$）.

	SHAM	SCI+NS	SCI+TPTD	SCI+ALN	F- value	P-value
trabecular BMD	0.8417±0.0460	0.7060±0.0380*	0.8552±0.0577#	0.7086±0.0423*	24.64	<0.001
cortical BMD	1.3870±0.0347	1.3170±0.0542*	1.3930±0.0316#	1.3720±0.0428	5.514	0.0042
BMC	9.2330±4.6420	2.4440±1.0340*	8.8090±4.0630#	3.3050±1.3510*	8.989	0.0002
BV/TV	31.81±15.45	7.788±3.276*	28.65±14.00#	12.66±4.971*	9.462	0.0002
BS/BV	23.79±2.901	17.98±3.333*	24.44±4.537#	20.02±5.016	4.640	0.0093
BS/TV	5.270±2.516	1.898±0.7064*	4.369±1.820#	3.389±1.400	5.512	0.0042
Tb.Th	0.1984±0.0362	0.1441±0.0162*	0.2049±0.0311#	0.1428±0.0271*	11.12	<0.0001
Tb.N	1.962±0.5825	0.7845±0.2666*	1.682±0.5442#	0.9359±0.1810*	14.07	<0.0001
Tb.Sp	0.3192±0.1153	0.5454±0.0497*	0.3636±0.0847#	0.5040±0.0614*	14.16	<0.0001
Ct.Ar	7.769±1.261	7.797±1.145	7.219±0.8703	7.593±0.8895	0.5089	0.6794
Ct.Th	0.5974±0.0239	0.5414±0.0276	0.6749±0.0694#	0.6611±0.0788#	9.824	0.0001
Ma.Ar	9.280±1.314	7.419±0.5730*	8.050±1.486	8.783±0.5763	4.651	0.0092

Comparison with SHAM group，*P<0.05；Comparison with SCI+NS group，#P<0.05.
